# Be Honest: Individuals’ Moral Responsibility within the COVID-19 Context

**DOI:** 10.21315/mjms2020.27.6.13

**Published:** 2020-12-29

**Authors:** Yusrita Zolkefli

**Affiliations:** PAPRSB Institute of Health Sciences, Universiti Brunei Darussalam, Brunei Darussalam

**Keywords:** truth, pandemic, COVID-19, health professionals, patients

## Abstract

We recognise that people lie to health professionals for several reasons. However, these incidents endanger the well-being of the professionals and bring us to the question of whether people have an exclusive moral duty to always profess the truth about their health and other facts, particularly in a pandemic crisis. This review argues that an honest patient is a key to undertaking their roles as health professionals and delivering the best services possible to meet the needs of the patient. Greater awareness and comprehension of the potential ramifications of dishonesty, not only helps establish the moral obligation, to tell the truth, particularly in a pandemic situation, but also translates into a better relationship with health professionals. It also enforces an ethical solidarity on every single of us to show tangible moral response to ensure that those most vulnerable to risks from the pandemic illness such as health professionals are protected as far as possible.

## COVID-19 Response

I read on the social media with complete dismay about how some people decided to conceal their history of travel or recent contact with suspected or confirmed COVID-19 cases. An example is a Malaysian patient who lied about contact with a participant of a *tabligh* (an Islamic mass gathering) and subsequently tested positive for COVID-19 and whose anaesthesiologist was then forced to quarantine for two weeks while the entire hospital had to be closed to carry out ‘terminal disinfection and fumigation’ (1). A study on honest disclosure suggests that there is a degree of concealment during the COVID-19 pandemic that may place the public and others at risk (2). We understand that people lie for many reasons; however, these incidents threaten health professionals’ safety and lead us to the question of whether there is an exclusive moral obligation for people to always being honest about their health and other information, particularly in a pandemic crisis.

## Reasons for Concealing Information

We can only speculate about various reasons for non-disclosure in a pandemic event. For example, it is most likely that any person suspected of having COVID-19 will have to undergo a multiple nasal swab tests, which may cause discomfort. In addition to its physical strain on patients and healthcare facilities, COVID-19 has considerable psychosocial effects (3). Hospital admission, for example, is a possible deterrent. If no one is available to care for their children, a single parent may choose to hide recent travel history, as hospitalisation or quarantine could result in more undesirable burdens on the loved ones (4). This leads one to argue that forcing information disclosure on someone who may not be prepared to deal with its impact — such as mandatory self-quarantine — may be perceived as unjustified if he or she ‘considers’ the risk of becoming infected to be negligible, while more serious issues may arise from being honest. This implies that the psychosocial impact may be too much for individuals to provide honest information, at least until there is a certainty that the well-being and safety of their loved ones will be protected. Furthermore, it is also possible that since Coronavirus is still a novelty, it remains debatable whether any information can be revealed to health professionals until one is certain that they have it. In general, hiding information from particular health professionals and not telling them honest information for a reason, such as privacy, is viewed as justifiable. For some, quarantine means being locked up somewhere, which also means that they may not be able to leave when they want. Arguably, the quarantine is often too late to effectively stop and control the pandemic (5). Moreover, quarantines are not without human cost. The Wuhan lockdown, for example, had many unintended effects, some of them fatal. People in the city could not reach sick, elderly parents, let alone take them elsewhere to treat chronic conditions like heart disease and cancer.

## Being Honest

The possible explanations for concealment presented above, are, however, problematic. Given the nature of the crisis, it must be argued that people have a fundamental and especially moral obligation to being honest about the risk of being infected with the virus. There should be no negotiation on the value of honesty, particularly when health professionals require the information in the best interest of all. The primary duty of health professionals is to help patients and not harm them; therefore, the same duty should be shared by patients in return, that is, they have the critical task of cooperating with and not harming health professionals, especially when these professionals are at the frontline of battling a pandemic. Thus, this type of dishonesty is generally not accepted because it causes further harm in a wider context to health professionals and other individuals. Kant (6), one of the great Western philosophers, argued that dishonesty and lying even in the most justifiable circumstances is wrong because we cannot be certain about the implications of our acts. In many ways, this premise strongly insinuate that telling the truth is a moral obligation (7), and lies ultimately becomes self-defeating, as people understand that they cannot depend on the words of others (8). It is no surprise that health professionals such as medical doctor and nurses expressed disappointment, realising that other individuals are merely acting selfishly in retaining key information. Public interest takes precedence over individual rights when it comes to public safety. It has to be understood that public accountability is an ethical principle according to which individuals are accountable for their public duty; the actions of an individual will benefit the community as a whole. In other words, it would be socially irresponsible if an action was detrimental to society or culture. Moral ideals inherent in culture differentiate right from wrong. A person must act in a way that benefits the society and not just the individual. Kant (6) believed that even though it may be dangerous, everyone has a strict responsibility to always be honest, as he felt that deception is never supposed to be an excuse, since it is always deleterious to one person or the society as a whole. Kant also believed that it is always a responsibility to speak the truth, whether it concerns the right of others to know or does significant harm to innocent people. In other words, from a deontological point of view, health professionals should be told the truth whatever the ramifications are.

In ethics, lying and being dishonest to health workers is considered an infringement of their autonomy as a person and a contradiction of concepts such as therapeutic relationships and partnership care. This is of fundamental importance for a patient seeking healthcare, and relevant health status or personal information must be disclosed truthfully. Appropriate and genuine information is needed by health professionals to prepare and deliver the highest-quality, accurate and evidence-based treatment; without genuine knowledge, health professionals cannot provide successful care. Moreover, in terms of psychological aspects, it is almost immoral (regardless of how trivial) to expose someone (in this case a health professional) to danger when they have the best intentions to help and support the society. The fact that a person does not reveal correct information after being swabbed and quarantined by health professionals and after the closing of the affected area of practice is damaging and an example of immoral behaviour and lack of responsibility, which can potentially contribute to health professionals’ significant psychological distress. The pandemic itself has generated a high degree of uncertainty and vulnerability that poses a danger to these professionals’ mental health (9). It is also troubling that the scarcity of personal protective equipment raises the likelihood of contracting the virus. Health professionals may also fear of spreading the virus to their loved ones, particularly the elderly or people with co-morbid illnesses. It is, therefore, fair to assume that one of the tenets of reality, particularly in this pandemic crisis, is respect for the health professionals as an individual with full rights to be treated with respect and not be harmed while on duty. They want nothing less than honest and truthful information about and from patients. Non-disclosure of honest information would not only prevent a patient from obtaining appropriate treatment without full knowledge of the facts, but would also put health professionals at risk of harm. There is, therefore, a need for a stronger emphasis on the responsibility of all parties, and not just governments, to take concerted action to ensure that health professionals are protected from harm in order to truly uphold human rights (10) and thus to enable the provision of highest standard of care to patients.

Given these common threats, the principle of solidarity negates collective action. It also fosters efforts to address inequalities that are impending welfare. Patients have healthcare obligations, and the concept of moral responsibility for specific others and the community can justify many of these obligations. Emergencies in public health highlight the need for unity-remembering what we owe each other as fellow, fair human beings. Although COVID-19 is likely to cause only mild disease for many people, a large minority of the population will suffer severe illness, as a result of which some may die. This sets an ethical duty on everyone to take seriously measures such as engaging in honest communication, not necessarily for personal gain, but to ensure that those most vulnerable to serious illness are covered as much as possible from infection (11). In fact, in moments of crisis, the individual has a duty not only to offer moral support to health professionals but also to support their overall safety and well-being. If this does not occur, it can result in a crippled healthcare system, which unfortunately puts vulnerable patients at risk of not receiving proper care. Health professionals have a wider duty to protect people against prospects of significant harm (12). Furthermore, concealing COVID-19 details will only hinder public health efforts to slow down COVID-19 spread and threaten the health of community members (2).

## The Way Forward

People who look to health professionals for support should always be honest and tell the truth. An honest patient is crucial to health professionals’ performing their duties and providing the best treatment available to meet the patient’s needs. Improved awareness and comprehension of the wider consequences of dishonesty not only helps create the moral responsibility of telling the truth, specifically in a pandemic situation, but also translates into a positive relationship with health professionals. The actual pandemic is a wake-up call for all of us. The lessons from past pandemic ethics are not to be ignored. Honesty is a crucial moral good, and it makes logical sense in the present circumstances of the COVID-19 pandemic that the utilitarian principle of the greatest good for the greatest number of people has more weight than a perceived concern for an individual’s psychosocial harm. It must be realised that COVID-19 is about having moral responsibility for every single one of us. Thus, having considered the arguments that obliging honest communication, particularly honesty in information disclosure is ethically justifiable, especially during a pandemic situation where individuals always have a strong and special moral responsibility to be honest in the disclosure of information.

## References

[1] Raj MR (2020). Kelantan doctor furious after patient lies about COVID-19 exposure to get MC. Malaymail.com.

[2] O’Connor AM, Evans AD (2020). Dishonesty during a pandemic: the concealment of COVID-19 information. J Health Psychol.

[3] Dubey S, Biswas P, Ghosh R, Chatterjee S, Dubey MJ, Chatterjee S (2020). Psychosocial impact of COVID-19. Diabetes Metab Syndr.

[4] Rush KL, Kjorven M, Hole R (2016). Older adults’ risk practices from hospital to home: a discourse analysis. Gerontologist.

[5] Wilder-Smith A, Freedman DO (2020). Isolation, quarantine, social distancing and community containment: pivotal role for old-style public health measures in the novel coronavirus (2019-nCoV) outbreak. J Travel Med.

[6] Kant I (1964). The doctrine of virtue.

[7] Bok S (1973). Lying moral choices in the public life and private life.

[8] Rachels J, Rachels S (2019). The elements of moral philosophy.

[9] Chen Q, Liang M, Li Y, Guo J, Fei D, Wang L (2020). Mental health care for medical staff in China during the COVID-19 outbreak. Lancet Psychiatry.

[10] Sikkink K (2020). Rights and responsibilities in the coronavirus pandemic.

[11] Nuffield Council on Bioethics (2020). Ethical considerations in responding to the COVID-19 pandemic.

[12] General Medical Council (2020). Disclosures for the protection of patients and others.

